# The Role of CD40, CD86, and Glutathione S-Transferase Omega 1 in the Pathogenesis of Chronic Obstructive Pulmonary Disease

**DOI:** 10.1155/2022/6810745

**Published:** 2022-08-23

**Authors:** Desheng Sun, Rong Lin, Yao Ouyang

**Affiliations:** Department of Respiratory and Critical Care Medicine, Affiliated Hospital of Zunyi Medical University, Zunyi, China

## Abstract

**Objective:**

The aim of the study was to explore the relevance of CD40, CD86, and GSTO1 with the pathogenesis of COPD.

**Methods:**

Patients with acute exacerbation of COPD were contrasted with the healthy and nonsmoking ones and smoking but without COPD ones. The changes of CD40, CD86, and GSTO1 in the peripheral blood, collected from different groups, were detected by flow cytometry and western blotting, respectively.

**Results:**

Compared with the nonsmoking group and smoking but without the COPD group, the expression of CD40 and CD86 of the patients with COPD increased significantly, but the expression of GSTO1 decreased. CD40 and CD86 were negatively correlated with FEV1%, while GSTO1 was positively correlated with FEV1% and negatively correlated with CD40 and CD86.

**Conclusion:**

CD40, CD86, and GSTO1 may play a role in the pathogenesis of COPD, and they are related to the severity of COPD and the degree of changes in the lung function.

## 1. Introduction

Chronic obstructive pulmonary disease (COPD) is a persistently airway-obstructed disease, characterized by irreversible airflow limitation and the progressive lesion of pulmonary function. WHO predicted that COPD will affect nearly 400 billion people and become the third leading cause of death worldwide until 2030 [1, 2]. Currently, several studies concluded that these factors, including pulmonary inflammation, oxidation-antioxidation imbalance, and protease-antiprotease imbalance, correlate with the pathogenesis of COPD; however, the precise mechanisms leading to COPD remain undefined.

Cigarette smoking is the primary risk factor for the development of COPD, and moreover, as the main source of an oxidant in pulmonary function, it results in the destruction of the antioxidation defense system, which puts smokers at a great risk of developing COPD. It has been reported that every puff of a cigarette inhales 10^15^ oxygen free radicals, while more neutrophils and macrophages are present in smokers' alveolar, further increasing the release of free radicals, which eventually inactivates the antiprotease. Besides, other than directly damaging the lung matrix components such as elastin or collagen, oxides also interfere with the synthesis and repair of elastin, generating emphysema [3].

Glutathione (GSH), a major antioxidant in airways, can be divided into two types: glutathione (GSH) and glutathione disulfide (GSSG). They form an effective antioxidant circulatory system and are able to maintain a certain proportion *in vivo* through the oxidation-reduction reaction to transform reciprocally between each other [4]. Research has demonstrated that glutathione S-transferase omega (GSTO) found in mammals, which belongs to glutathione S-transferase (GST), has potential effects on regulating the homeostasis of GSH. Several oxidant free radicals are electrophilic compounds or converted into electrophilic after being metabolized by cytochrome enzyme P450 in a cigarette. Such substances are used as substrates by GSTO to catalyze the combination with GSH for detoxification. Furthermore, GSTO also contains an N-terminal glutathione binding domain that enables it to promote metabolism and maintain GSH levels in damaged cells. Therefore, we detected the expression of GSTO1 in COPD patients.

Notoriously, smoking has been found to cause chronic inflammation of the airways, then leading to irreversible restriction of expiratory flow and progressive decrease of pulmonary function [5]. However, accumulating evidence has identified that chronic bronchial inflammation in COPD patients continues to develop after they quit smoking, suggesting that immune factors may also participate in the pathogenesis of COPD [6]. Dendritic cells (DCs), the most potent professional antigen-presenting cells (APCs), play a vital role in initiating, regulating, and maintaining specific immune responses [7, 8]. Our previous animal experiments found that DC may affect the occurrence of COPD; the markers of dendritic cells in the rat model of COPD induced by smoking increased significantly [9]. Therefore, in this study, we also detected CD40 and CD86 markers of dendritic cells in patients with COPD. In this study, we primarily explored the relevance of CD40, CD86, and GSTO1 with the development of COPD.

## 2. Materials and Methods

### 2.1. Patients and Healthy Individuals

Peripheral blood samples were acquired from 29 COPD patients (21 males and 8 females, range 55–82 years), including 10 cases with COPD GOLD I(7 males and 3 females with a median age of 66.20 ± 7.42 years), 8 cases with COPD GOLD II (6 males and 2 females with a median age of 69.25 ± 7.78 years), and 11 cases with COPD GOLD III-IV (8 males and 3 females with a median age of 67.98 ± 6.93 years) and 17 healthy individuals (11 males and 6 females, range 55–76 years) as healthy controls whose race, sex, and age were roughly the same as those of COPD patients, including the nonsmoking group (6 males and 3 females with a median age of 65.89 ± 6.75 years) and smoking but without the COPD group (5 males and 3 females with a median age of 67.75 ± 6.04 years) as shown in Table 1. In particular, all of the COPD patients in this study have a long history of smoking.

All examiners extracted 2 ml of fasting elbow venous blood in the early morning and put it in an EDTA anticoagulative tube. Among them, samples from COPD patients were collected on the day after admission. We excluded COPD patients and healthy controls with other hematological, autoimmune diseases, or infections. All samples were obtained with consent. The diagnostic basis of COPD was in compliance with the Diagnostic Guidelines for Chronic Obstructive Pulmonary Disease (COPD). In addition, the study was approved by the Ethics Committee of the Affiliated Hospital of Zunyi Medical University. Written informed consent was obtained from patients in accordance with the Helsinki Declaration.

### 2.2. Flow Cytometry Analysis

In flow cytometric analyses, nucleated cells were obtained by red blood cell lysis of blood from individuals. Cells were incubated with specific antibodies directed at surface markers including PE mouse anti-human CD86 and FITC mouse anti-human CD40. Then, the cells were washed and centrifuged (1000 rpm for 5 min) to prepare for detecting the CD40 or CD86 percentages in the different cell subsets. 1% paraformaldehyde was used to fix them. Isotype controls were supplied to enable compensation and confirmation of antibody specificity. The values of flow cytometry were recorded and analyzed using a flow cytometer (Becton, Dickinson and Company, New Jersey, USA) by the Cell Engineering Laboratory of Affiliated Hospital of Zunyi Medical University.

### 2.3. Western Blotting

Immunoblotting was performed on peripheral blood plasma extracts. Harvested proteins were lysed at room temperature, then using ddH_2_O to attenuate it 20 times. By using a transfer system (BIO-RAD, California, USA), we transfered the proteins to a PVDF membrane for 4 h for 40 V in ice bath conditions. Subsequently, to impede nonspecific protein-binding sites on it, the PVDF membranes were washed in TBST for 30 min. The membranes were incubated overnight with primary antibodies against GSTO1 and *β*-actin (both from Abcam, Los Angeles, USA). After washing with Tris-buffered saline and Tween 20 solution, membranes were incubated with secondary antibodies for 1 hour. The signal was quantitated using Odyssey Scanning Imaging System (LI-COR, Lincoln, USA).

### 2.4. Data and Statistical Analyses

The data were carried out using SPSS (version 19.0; SPSS InC., Chicago, IL, USA) and represented as the mean ± standard deviation (SD). One-way analysis of variance was used to compare statistics among multiple groups, and comparisons between two conditions were performed with LSD or Tamhane. Correlations were measured with Spearman correlation analysis. Values of *P* < 0.05 were considered statistically significant.

## 3. Results

### 3.1. Pulmonary Function of Different Groups

Compared with the nonsmoking group and smoking without the COPD group, the lung function indexes (FEV1% and FEV1/FVC) were significantly lower in COPD patients. Moreover, with the upgrade of the GOLD classification, this trend is more obvious. In other words, the lung function of GOLD II patients was significantly lower than that of GOLD I patients, and lung function of GOLD III-IV patients was significantly lower than that of GOLD II patients (Table 2).

### 3.2. Flow Cytometry Results of CD40 and CD86

**Figure 1:**
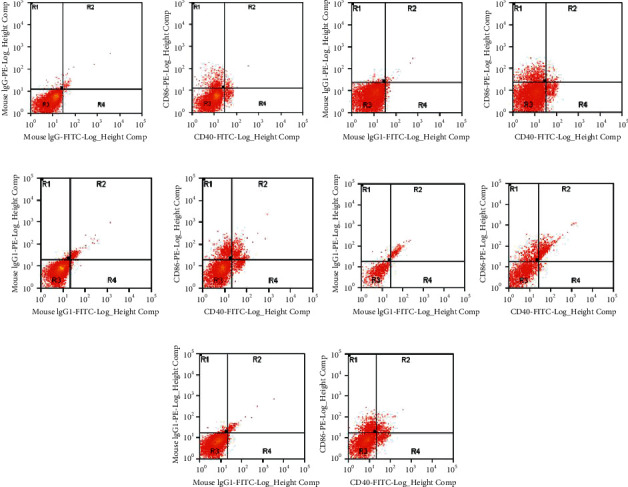
The results of CD40 and CD86 in healthy individuals and COPD patients. The expression of CD40 and CD86 were analyzed by FCM (left for the control tube, right for the test tube), including nonsmoking group (a), smoking but without COPD group (b), GOLD I group (c), GOLD II group (d), and GOLD III-IV group (e).

The CD40 and CD86 levels of peripheral blood were measured in different populations using flow cytometry (FCM). There was a significant accumulation of the expression of CD40 or CD86 in COPD groups compared with controls (Figure 1, Table 3).

### 3.3. GSTO1 Protein Expression of Different Groups

**Figure 2:**
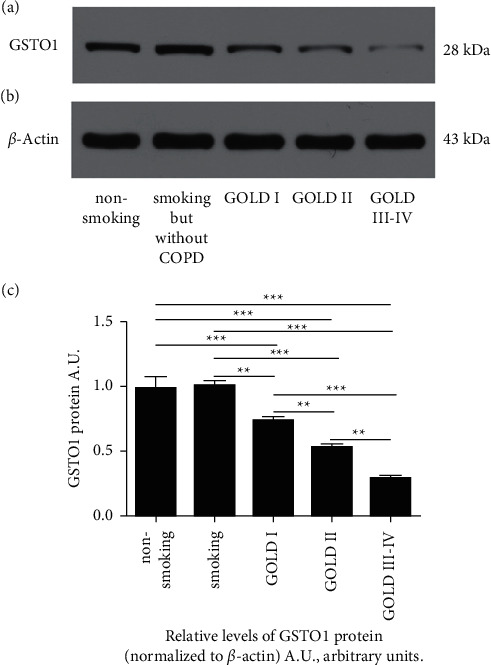
The expression of GSTO1 in blood from different groups of subjects. Representative bands of protein expression for GSTO1 (a) and *β*-actin (b). (c) Comparison of relative GSTO1 protein levels (normalized to *β*-actin level) among the different groups. ^*∗∗*^*P* < 0.01, ^*∗∗∗*^*P* < 0.001.

Immunoblot analysis of peripheral blood isolated from individuals was conducted to detect GSTO1 protein levels, demonstrating that the expression of GSTO1 was dramatically decreased compared to controls (Figure 2).

### 3.4. The Correlation between GSTO1 and CD40 and CD86 and Their Correlation with the Lung Function

**Figure 3:**
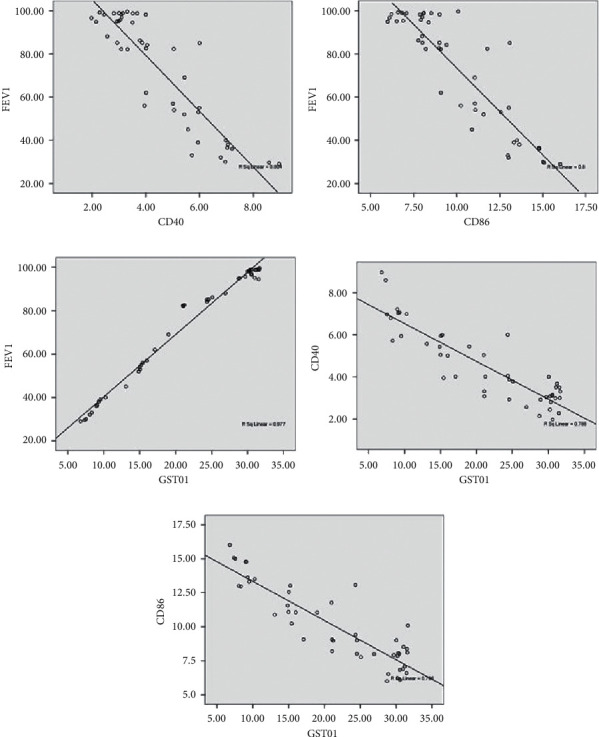
The correlation between CD40, CD86, and GSTO1 and their correlation with FEV1%, respectively. Correlations between FEV1% and CD40 (a) or CD86 (b). (c) Correlations between GSTO1 and FEV1%. Correlations between GSTO1 and CD40 (d) or CD86 (e).

To evaluate the association of DCs and the lung function, we hence analyzed the correlation between CD40 or CD86 percentages and the forced expiratory volume in one second (FEV1). The analysis result showed the significant negative correlation between the content of DCs and pulmonary function (CD40 vs. FEV1%, *r* = −0.897, *P* < 0.05; CD86 vs. FEV1%, *r* = −0.894, *P* < 0.05; Figures 3(a) and 3(b)).

Meanwhile, positive correlations were observed between the GSTO1 level and FEV1% (*r* = 0.989, *P* < 0.05; Figure 3(c)). Besides, the evident negative correlation between CD40 or CD86 percentages and GSTO1 was also found (CD40 vs. GSTO1, *r* = −0.888, *P* < 0.05; CD86 vs. GSTO1, *r* = −0.892, *P* < 0.05; Figures 3(d) and 3(e).

## 4. Discussion

COPD is a frequently occurring disease, characterized by airflow limitation and the progressive lesion of pulmonary functions. Smoking is the most common risk factor for COPD. Smoking causes injury to the human body through actions of multiple factors, including oxidative stress, airflow obstruction, and disruption of protease-antiprotease balance. It is reported that the decline of pulmonary function in COPD patients is related to smoking [10], and our research compared pulmonary function between different groups. We demonstrated that FEV1% and FEV1/FVC were decreased in COPD groups compared with controls (including the nonsmoking group and smoking but without the COPD group). Relatively, these parameters were descended slightly in smoking but without the COPD group compared with the nonsmoking group. There was no statistically significant difference in pulmonary function between these two groups, which may be due to the less sample size and the impact on the statistical results from the smoking cumulants, patterns, and time.

Plenty of oxygen free radicals in cigarette smoke increase the burden of oxidation *in vivo* of smokers or even result in oxidative stress, favoring the severe damage to the body. Through the research of 8-iso-prostaglandin (8-iso-PG) in serum, some scholars noticed that smokers without COPD were also shown to have markedly elevated this index compared with nonsmoking people, suggesting that smoking certainly increases the oxidative burden of the body [11], which is of great significance for the vast majority of COPD patients who smoke. Oxidative stress directly destroyed the airway epithelium and aggravated the inflammatory response, while the protease-antiprotease balance was disrupted, resulting in continuous restricted airflow ultimately. In summary, using induced sputum, peripheral blood, and other specimens from COPD patients, scholars demonstrated that the indicators of oxidation such as malondialdehyde (MDA), 8-iso-PG, and GSSG grew obviously. Conversely, indexes that can reflect antioxidant capacity, such as GSH and superoxide dismutase (SOD), were significantly decreased. Meanwhile, the degree of oxidative stress was correlated with pulmonary function [12]. However, another study on COPD rats and patients found an increased level of GSH in COPD groups compared with controls, whereas the reactive oxygen species (ROS) were increased more dramatically. These findings suggest that although GSH increased, its antioxidant capacity attenuated, triggering oxidation-antioxidation imbalance [13].

The occurrence of oxidative stress is not only created by the aggravation of oxidation degree but also can be caused by (or accompanied by) the reduction of antioxidant capacities, such as the reduction of antioxidants, antioxidant enzymes, or their antioxidant capacity. GSH, a major antioxidant in the pulmonary function, can be divided into two types: GSH and GSSG. In physiological conditions, they form an effective antioxidant circulatory system and are able to maintain the dynamic balance in vivo through glutathione peroxidase (GSH-Px) and glutathione reductase (GSH-R) to transform mutually from each other. Studies have shown that GSTs are involved in about 50% GSH-Px activity. Furthermore, GSTO contains an N-terminal glutathione binding domain that may enable it to promote the metabolism and maintain the level of GSH, which acts as a potential reservoir during oxidative stress and may also regulate the structure of GSH protein. Currently, the research on GSTs mainly focuses on their relationship with tumors and the gene polymorphism of disease, etc. Our findings demonstrated that GSTO1 was significantly reduced with COPD exacerbation in patients. Meanwhile, the GSTO1 level is positively correlated with pulmonary function, indicating that there was oxidative stress in COPD patients, which is closely associated with pulmonary function. Besides, there was no apparent difference in GSTO1 between nonsmokers and smokers without COPD, so further investigation into that whether GSTO1 is associated with smoking is needed.

There is growing evidence that chronic bronchial inflammation in COPD patients continues to develop after they quit smoking, indicating that immune factors may participate in the pathogenesis of COPD. DC, derived from hematopoietic stem cells in the bone marrow and monocytes in peripheral blood [14], is the only antigen-presenting cell that can activate the proliferation and activation of original T cells, which is positioned to perform a crucial role in the connection between specific and nonspecific immunity. The role and changes of DCs in COPD have been widely concerned, but as to its quantity and function, there are controversial conclusions. Through the examination of COPD amounts, Rogers et al. found that compared with patients who had quit smoking, the number of DCs in the bronchial mucosa of COPD patients was reduced, while the former had no significant difference compared with nonsmokers [15]. In Tsoumakidou's study, the quantity of mature DCs (mDCs) decreased in the airways of smokers without COPD, and an increase in immature DC (iDC) amounts was found in the small airway of COPD patients. Also, total DCs in the airways of smokers with COPD were decreased [16]. Demedts et al. found that compared with healthy nonsmokers and smokers without COPD, the number of airway DCs in COPD patients was significantly increased, but there was no difference in the number of airway DCs between smokers and nonsmokers [17]. Su et al. used immunohistochemistry to detect the changes of DCs in the lungs of the population and found that compared with nonsmokers, the DCs in the lungs of COPD patients increased significantly, and the difference was statistically significant. Compared with smokers without COPD, the levels of DCs in COPD patients have increased, but the difference is not statistically significant [18].

Normally, the expression of cluster of differentiation 40 (CD40) and cluster of differentiation 86 (CD86) was at a sustainable low level on the DC surface. However, their levels will significantly increase when the body suffers from inflammation and other stimulation. In the current study, our data supported that CD40 and CD86 were involved in the occurrence and development of COPD, and the increase of DC in peripheral blood of COPD may be conducive to its recruitment to pulmonary tissues, further participating in the pathogenesis of COPD. On the flip side, respiratory disease such as COPD mainly affects the lungs and can also cause damage to multiple systems. Nevertheless, there was no outstanding difference in CD40 or CD86 percentage of the smoking group without COPD compared with that of the nonsmoking group, attesting that related factors may influence the results of the experiments, or smoking alone led to no significant effect on DCs. Meanwhile, our analysis results showed a significant negative correlation between CD40 or CD86 percentage and pulmonary function (FEV1), which further indicated that DCs perform a vital role in the pathogenesis of COPD.

Although some studies suggest that oxidative stress and dendritic cells may play a role in the occurrence and development of COPD, there are few joint studies on the two in COPD. DCs will mature into mDCs after specific stimulation. Also, such mDCs are generated under the irritation of oxidative stress, smoking, lipid abnormalities, and pathogeny microbe infection [19]. Wang et al. established a murine model of asthma and found that oxidants stimulated CD40 and CD86 into high expression, but their content decreased after preventing oxidative stress [20].

It can be seen from the above studies that the increase of oxidants in many diseases can promote the maturation of DCs. Does the same mechanism also exist in COPD? Does the weakening of antioxidant capacity also stimulate the maturation of DCs? In our experiments, after analyzing the correlation between GSTO1 and DCs, we found that these two have a negative correlation; that is, the maturation of DCs is related to oxidative stress, and the weakening of antioxidant capacity can stimulate iDCs to mature. With the degree of oxidative stress exacerbated, the number of mDCs (to be exact, its markers, CD40 and CD86) increased significantly, which may be due to the DCs' response to oxidative stress as a “danger signal.”

The most important limitation of our research was the relatively small sample size, which limits forming strong conclusions, and a larger study is needed. In addition, this study has not yet explored the potential molecular mechanism, which needs to be considered in the follow-up study.

## 5. Conclusion

Our study demonstrated that CD40 and CD86 are indicators of COPD, and GSTO1 may inhibit COPD. In addition, they are related to the severity of COPD and the degree of changes in the lung function. The specific mechanism needs further study.

## Figures and Tables

**Table 1:** Basic information of different groups.

	Nonsmoking	Smoking but without COPD	GOLD I	GOLD II	GOLD III∼IV
*N*	9	8	10	8	11
Sex (Male/Female)	6/3	6/2	7/3	6/2	8/3
Age (years)	65.89 ± 6.75	67.75 ± 6.04	66.20 ± 7.42	69.25 ± 7.78	70.55 ± 6.80

Note. There is no statistical significance in the comparison between groups.

**Table 2:** Measurement results of lung function in different groups.

	Nonsmoking	Smoking but without COPD	GOLD I	GOLD II	GOLD III∼IV
*N*	9	8	10	8	11
FEV1% (%)	98.32 ± 1.40	96.46 ± 1.69	84.28 ± 2.00^*∗*^^#^	57.25 ± 5.65^*∗*^^#^▲	35.28 ± 5.06^*∗*^^#^▲■
FEV1/FVC (%)	80.06 ± 1.49	79.35 ± 1.97	63.70 ± 3.06^*∗*^^#^	52.85 ± 1.32^*∗*^^#^▲	41.65 ± 1.86^*∗*^^#^▲■

^*∗*^Compared with the nonsmoking group, *P* < 0.01; ^#^compared with smoking but without the COPD group, *P* < 0.01; ▲compared with the GOLD I group, *P* < 0.01; ■compared with the GOLD II group, *P* < 0.01.

**Table 3:** Expression of CD40 and CD86 in different groups.

	Nonsmoking	Smoking but without COPD	GOLD I	GOLD II	GOLD III∼IV
*N*	9	8	10	8	11
CD40 (%)	2.99 ± 0.63	3.01 ± 0.47	3.87 ± 1.03^*∗*^^#^	5.10 ± 0.78^*∗*^^#^▲	6.98 ± 1.06^*∗*^^#^▲■
CD86 (%)	7.53 ± 1.37	7.55 ± 0.92	9.33 ± 1.75^*∗*^^#^	11.21 ± 1.24^*∗*^^#^▲	13.91 ± 1.41^*∗*^^#^▲■

^*∗*^Compared with the nonsmoking group, *P* < 0.05; ^#^compared with smoking but without the COPD group, *P* < 0.05; ▲compared with the GOLD I group, *P* < 0.05; ■compared with the GOLD II group, *P* < 0.05.

## Data Availability

The data that support the findings of this study are available from the corresponding author upon reasonable request.
